# Who wants to be involved in health care decisions? Comparing preferences for individual and collective involvement in England and Sweden

**DOI:** 10.1186/s12889-017-4534-y

**Published:** 2017-07-14

**Authors:** Mio Fredriksson, Max Eriksson, Jonathan Tritter

**Affiliations:** 1 Department of Public Health and Caring Sciences, Uppsala University, Box 564, 751 22 UPPSALA, Sweden; 2 School of Languages and Social Sciences, Aston University, Birmingham, B4 7ET UK; 3 Department of Political Science, Umeå University, 901 87 Umeå, Sweden

**Keywords:** Patient and public involvement, Sweden, England, Local organisation decisions, Treatment decisions

## Abstract

**Background:**

Patient and public involvement (PPI) is framed as positive for individuals, the health system, public health, as well as for communities and society as a whole. We investigated whether preferences for PPI differed between two countries with Beveridge type health systems–Sweden and England. We measured willingness to be involved in individual treatment decisions and in decisions about the organization and provision of local health and social care services.

**Methods:**

This was a comparative cross-sectional study of the general population’s preferences. Together, the two samples included 3125 respondents; 1625 in England and 1500 in Sweden. Country differences were analysed in a multinomial regression model controlling for gender, age and educational attainment.

**Results:**

Overall, 68% of respondents wanted a passive patient role and 44% wanted to be involved in local decisions about organization and provision of services. In comparison with in Sweden, they were in England less likely to want a health professional such as a GP or consultant to make decisions about their treatment and also more likely to want to make their own decisions. They were also less likely to want to be involved in local service development decisions. An increased likelihood of wanting to be involved in organizational decision-making was associated with individuals wanting to make their own treatment decisions. Women were less likely to want health professionals to make decisions and more likely to want to be involved in organizational decisions.

**Conclusions:**

An effective health system that ensures public health must integrate an effective approach to PPI both in individual treatment decisions and shaping local health and social care priorities. To be effective, involvement activities must take in to account the variation in the desire for involvement and the implications that this has for equity. More work is needed to understand the relationship between the desire to be involved and actually *being* involved, but both appear related to judgements of the impact of involvement on health care decisions.

## Background

Patient and public involvement (PPI) in healthcare is an increasingly important aspect of European health systems [1–5]. Involvement is framed as positive for individuals, the health system, public health, as well as for communities and society as a whole [6]. Yet, in the European context we have insufficient knowledge of who and to what extent people want to be involved in healthcare decisions, and whether this varies by individual characteristics or differs between countries. In this article we measure the wish to be involved in health care decisions in two countries with Beveridge type health systems – England and Sweden. The core principles of Beveridge systems are universality and equity (e.g. Denmark, Finland, Greece, Ireland, Italy, Norway, and Spain), in contrast to systems of Bismarck-type that are built on the principles of plurality, liberty and solidarity (e.g. Austria, Belgium, France, Germany, Netherlands, and Switzerland). Importantly, Beveridge systems are funded by means of general taxation while Bismarck systems are funded by means of earmarked premiums, mainly from salaried employees [7, 8]. In these two countries, we investigate preferences for involvement in two types of decisions or activities; individual treatment and local service development. Individual treatment decisions are linked to the perspective of the health service user (the patient) focusing on decisions about one’s own care. Strategic decisions about health services and policy at local or national level are associated with the perspective of a *public policy agent* (member of the public) and take account of a broader public interest [4, 9].

Earlier research elucidating preferences for involvement in healthcare has focused on the health service user’s preferences for involvement in treatment decisions, and shown significant variability in how much patients want to participate in individual decisions [10–12]. For example, it has been shown that 69% of patients with long-term conditions preferred to leave their medical decisions to their physicians [13] and that as few as 1.2% wanted an autonomous role [14]. Thus, evidence in the literature is inconclusive leading Nota et al. to conclude that “it remains difficult to explain or predict patient preferences” [15]. Most studies investigate involvement preferences in restricted patient populations rather than in the general population; one exception being Levinson et al. [16]. From a population-based study in the U.S they concluded that 52% preferred the physician make the decisions (characterised as a passive patient role by Arora and McHorney [13]). Women, more educated and healthier individuals were less likely to prefer the physician to make the decisions. Up to the age of 45 years the preferences for an active role increased.

There has been far less research on individual preferences for involvement in strategic decisions about health services and policy; the role fulfilled by the public policy agent. An annual survey in one of the 21 Swedish regions found that 64% of the residents thought the public should participate in prioritization discussions [17]. Similarly, in the UK, a focus group study showed that people expressed a strong desire for the public to be involved in different types of rationing decisions, but evidence of the willingness to be involved at individual level is much less explicit [18]. It may be that people are more interested in being involved in relation to some types of healthcare intervention such as organ donation or end of life care [19]. Anecdotal evidence from public involvement activities suggest that those individuals who actually get involved are “the usual suspects” [20] or the “vocal majority” [21]. It is not clear if the usual suspects are also more active in individual treatment decisions.

To investigate preferences, we gathered and compared data on the preferences of the general population in Sweden and England for different aspects of patient as well as public involvement. Specifically, (1) we sought to compare and contrast expressed preferences for individual decision-making in a medical setting with the desire to be involved collectively in the organisation and provision of local health care services in England and Sweden. Secondly (2) we sought to understand how the orientation towards acting locally was informed by expectations of such involvement leading to change, and finally (3) we sought to explore the implication of individual sociodemographic characteristics (gender, age and educational attainment) on reported preferences. No previous studies have investigated the desire to be involved in individual treatment decisions (the health service user perspective) and in local decision making concerning the organisation and provision of health and social care services (the public policy agent perspective) in the same individuals in a general population. The combination of these perspectives contributes to a more comprehensive understanding of the conditions for involvement in health; in individual health matters as well as matters of public concern. Encouraging patients to take more control when they are ill may prove to be an effective tool for improving public health [22] engendering the co-production of wellbeing, and empowering citizens and putting them at the “heart of the system” is one of the four core principles of the EU Health Strategy [23, 24]. For instance, in Sweden, participation and influence in society is seen as one of the fundamental conditions for public health. Public health policy in Sweden points out that a lack of influence or lack of opportunities for involvement in decisions that affect people’s living conditions and the development of societal functions are negatively correlated to health [25]. What is less clear is whether there is a link between the interest and likelihood of individuals to be involved in personal treatment decisions and to be involved in the development of local health services.

We chose to compare Sweden and England, countries which have similar health systems of Beveridge type. The English and Swedish health systems differ, however, in relation to the degree of decentralization and sub-national democratic governance, degree of patient centeredness and the set-up for PPI [26, 27]. Health system reform over the last 20 years in both countries has promoted markets and competition underpinned by a commitment to patient choice [28]. In Sweden, choice of provider in primary care (backed up by freedom of establishment for private providers) has been the central mechanism for strengthening the patient’s position since the mid-2000s [29, 30], from 2015 backed by the first patient law promoting the patient’s position, integrity, autonomy and participation (Patientlag 2014:821).

Although patient choice has also been at the top of the policy agenda in England [28], it is less apparent in practice as efforts to retain people’s confidence in the services provided have combined an economically motivated consumerist approach aiming at improving efficiency with ideologies of democratic public engagement [31]. Despite significant development of opportunities for involving both patients and the public through voice as well as choice mechanisms and to shape local health priorities under successive legislation in 2001 and 2007, these were changed by the Health and Social Care Act 2012. People are currently involved through local *Healthwatch* organizations (the consumer champion for health and social care [32]), the *NHS Citizen* (a national programme to give the public a say on healthcare matters and influence NHS England decision making [33]) or through local *patient participation groups* (PPGs); the latter a contractual requirement for all English primary care practices from 2015 which should also make reasonable efforts for PPGs to be representative of the practice population [34]. NHS England– that set the priorities and direction for the NHS – are also working to transform participation in health and care and help commissioners of health and care services to involve patients and carers in decisions relating to care and treatment and the public in commissioning processes and decisions. To involve the public, commissioners should, for example, develop joint approaches with local authorities, health and well-being boards, local Healthwatch, voluntary groups and other organisations, especially those who have existing relationships with local communities [35].

In comparison to England, public involvement in Sweden is more embedded in democratic institutions; the public primarily being represented by democratically elected representatives in the 21 healthcare regions, implying that healthcare decision-making is based on public interest [36]. Public involvement is generally framed as a way to influence the political process and political decisions within a region. To increase people’s opportunities to be involved between regional elections (between-election democracy) and in more practical aspects of service planning, more participatory forms of public involvement have gradually emerged gathered under the umbrella term “citizen dialogue” [37], which refers to mechanisms such as citizen surveys, panels or public meetings. Both Sweden and England have a long history of involving both patients and the public within similar Beveridgian health systems and their comparison provides an opportunity to investigate the patterns in preferences for involvement.

## Methods

### Study design

This was a comparative study of two cross-sectional nationally representative samples.

### Sample and procedure

On behalf of the authors, four questions were asked to a national random sample of members of the public in Sweden and in England, aged 15 and over. In England (*n* = 1625) the questions were asked as part of the Public Health England public opinion survey (2014) carried out by Ipsos MORI. In Sweden (*n* = 1500) the questions were part of an omnibus survey carried out by TNS Sifo. We used the weighting created by Ipsos MORI and TNS Sifo to account for sampling bias, see Table 1. In this article we present the results from three parallel questions, QI-Q3 (see Table 2). The questions were written in English, translated in to Swedish and back-translated in to English to validate wording.

**Table 1:** Survey characteristics

COUNTRY	Sample size	Data weighting: Nationally representative sample	Survey Method	Date	Survey conductor
ENGLAND	*n* = 1625 (age 15+)	Data weighted by gender, age, ethnicity, working status, social grade, housing tenure and Government Office Region (GOR) to be representative of the population.	Face-to-face omnibus survey	6–14 June 2014	Ipsos MORI
SWEDEN	*n* = 1500 (age 15+)	Data weighted by gender, age, working status and area code to be representative of the population.	Telephone omnibus survey	15–19 and 22–23 September 2014	TNS Sifo

**Table 2:** Survey questions

Q1	Please listen to the following pair of statements and decide, on a scale of 1 to 5, which comes closest to your own opinion. A score of 1 means you agree much more with statement A while a score of 5 means you agree much more with Statement B. A score of 3 means you agree equally with both, or don’t agree with either. (1) *Statement A* – *In general, I want a health professional, such as a GP or a consultant, to make decisions about my treatment*. (5) *Statement B – In general, I want to make my own decisions about my treatment, not rely on a health professional, such as a GP or consultant*. (6) Don’t know.
Q2	Please tell me whether on the whole you agree or disagree with the following statement: *I would like to be involved in decision making concerning the organisation and provision of health and social care services in my area.* (1) Strongly agree, (2) Agree, (3) Neither agree nor disagree, (4) Disagree, (5) Strongly disagree, (6) Don’t know.
Q3	Please tell me whether on the whole you agree or disagree with the following statement: *People (The public) in my area are able to help make improvements to health and social care services.* (1) Strongly agree, (2) Agree, (3) Neither agree nor disagree, (4) Disagree, (5) Strongly disagree, (6) Don’t know.

Approval for the use of the data in this article has been collected from Public Health England owning the English data collected via Ipsos MORI and from Uppsala University owning the Swedish data collected via TNS Sifo

### Measurements

Q1 was designed to investigate to what extent people (as health services users) want to be involved in decisions about their medical treatment. We measured one aspect of involvement: making the final decisions about treatment. Involvement may also refer to seeking and exchanging information, and discussing options in care [16]. In the literature, the wish for doctors to make the final decisions has been referred to as a passive role, a physician-directed approach or a paternalistic model [13].

Q2 was designed to investigate if people (as public policy agents) wish to be involved in decision-making about local health services. Q3 was designed to investigate whether people (as public policy agents) feel that they can influence decisions about the local health services.

Based on findings in previous research we included gender, age and educational attainment in the analysis. Gender (nominal) as well as age (continuous) was measured in the same way in the two datasets, whereas level of educational attainment was specified slightly differently due to differences in the educational systems. Data was collapsed into four categories: don’t know, low level of education (GCSE/O-level/CSE or similar), medium level of education (A-level or equivalent) and high level of education (minimum bachelor’s degree).

### Statistical analysis

Data analysis was done using Stata 13. Country differences were measured using χ2 and a significance level set at 95%. We employed a multinomial regression model using collapsed versions of Q1-Q3 as categorical outcome variables. Being neutral (alternative 3) was used as a base line category and relative odds ratios were calculated with respect to either agreeing (response alternatives 1 + 2) or disagreeing (response alternatives 4 + 5) with Q1-Q3. Response categories were combined in order to avoid possible central tendency biases arising from the Likert scale measurement (c.f. [38]) and to facilitate interpretations of the results found. When evaluating the effects of variables all other covariates were held constant at their respective means.

## Results

Together, the two samples included 3125 respondents, 52% of whom were English and 51% of whom were female. The average age was 51 years (SD 20). The proportion of respondents with a low level of education was 34%, medium level education 32% and high level of education 34%. Percentages for Q1-Q3 for both countries together are presented in Table 3.

**Table 3:** Percentages for Q1-Q3 in England and Sweden

Responses	Q1 N (%)	Q2 N (%)	Q3 N (%)
1	1485 (48)	577 (19)	339 (11)
2	547 (18)	773 (24)	773 (25)
3	620 (20)	828 (27)	1049 (34)
4	151 (5)	512 (16)	536 (17)
5	191 (6)	386 (12)	247 (8)
6 (Don’t know)	131 (4)	84 (3)	84 (6)
N	3125	3125	3125

The above percentages presented have been rounded

### Descriptive results

The distribution of Q1-Q3 when comparing Sweden and England showed a higher proportion of the population in Sweden agreeing to all three questions. In terms of willingness to make decisions about their own medical treatment (Q1) the two countries had similar proportions of respondents being neutral while respondents from Sweden were less neutral than the English respondents with regards to willingness to be involved in local decisions concerning the organisation and provision of health and social care services, as well as regarding people’s ability to help make improvements to health and social care services (Q2 and Q3). Respondents from the English sample showed a higher proportion that disagreed across Q1 and Q2, with overlapping confidence intervals with regards to Q1 (see Table 4).

**Table 4:** Descriptive presentations Q1-Q3

Responses	Q1: Individual decisions		Q2: Organizational decisions		Q3: able to make improvements	
	England	Sweden	England	Sweden	England	Sweden
Agree (%)	66 ± 2.4	70 ± 2.4	33 ± 2.3	55 ± 2.6	36 ± 2.4	39 ± 2.6
N	1005	1027	531	788	574	538
Neutral	21 ± 2.0	20 ± 2.1	30 ± 2.2	25 ± 2.2	40 ± 2.4	31 ± 2.5
N	319	301	475	353	626	423
Disagree (%)	13 ± 1.7	10 ± 1.5	37 ± 2.4	21 ± 2.1	24 ± 2.1	30 ± 2.4
N	197	145	595	299	377	406

### QI – Patient participation in treatment decisions

Excluding the don’t know-answers (1.7% in Sweden and 6.1% in England) a χ2-test suggests that there was an overall country difference in the preferred level of involvement in treatment decisions (χ2 = 22.6479, df (2), *p* = 0.001). In England 66% (± 2.4) answered that they want a health professional such as a GP or a consultant to make decisions about their treatment, compared to 70% (± 2.4) in Sweden. Furthermore, 13% (± 1.7) of the English sample responded that they want to make their own decisions and not rely on a health professional, compared to 10% (± 1.5) in Sweden.

### Q2 – People’s wish to be involved in organizational decision-making

Excluding the don’t know-answers (3.4% in Sweden and 1.5% in England) a χ2-test shows that there was a country difference in people’s wish to be involved in organizational decision-making (χ2 = 146.9667, df (2), *p* = <0.001). The Swedes were more positive towards being involved as 55% (± 2.6) agreed with the statement that they would like to be involved in local decision making concerning the organisation and provision of health and social care services compared to 33.0% (± 2.6) in England. Furthermore, 21% (± 2.1) of the Swedish disagreed compared to 37% (± 2.4) among the English.

### Q3 – Are people able to help improve services?

Excluding the don’t know-answers (9.5% in Sweden and 2.9% in England), a χ2-test suggests that there was an overall country difference in the perception of whether people are able to help make improvements to health and social care services at the local level (χ2 = 14.4990, df(2), *p* = 0.0067). The Swedes to a greater extent agreed with the statement that people are able to help make improvements (39% ± 2.6, compared to 36% ± 2.4 in England) as well as disagreed (30% ± 2.4, compared to 24% ± 2.1 in England).

### Country differences and sociodemographic characteristics

Controlling for gender, age and education, there were significant differences between England and Sweden regarding involvement in treatment decisions, as well as in regard to decisions about the organisation and provision of health and social care services (see Table 5). There were no significant differences between the English and the Swedish respondents in relation to whether people were able to help make improvements to local health and social care services.

**Table 5:** Country differences and sociodemographic characteristics

Response alternative	Independent variables	Dependent variables		
		Q1	Q2	Q3
Agree (1 + 2)	Age	.997 (.003)	1.015*** (.004)	1.007 (.004)
	Female	.787* (.088)	1.294* (.156)	.905 (.106)
	Low level of education	1.272 (.193)	.518** (.103)	.931 (.175)
	High level of education	1.013 (.134)	.998 (.135)	.881 (.115)
	England	.697** (.084)	.642** (.084)	.943 (.126)
	Q1	NA	1.285** (.101)	1.018 (.079)
	Q2	1.043 (.073)	NA	.684*** (.044)
	Q3	1.199 (.081)	.830** (.057)	NA
	Constant	3.314*** (.833)	.979 (.283)	1.674 (.490)
Disagree (4 + 5)	Age	1.004 (.004)	1.017*** (.004)	1.014*** (.004)
	Female	.961 (.156)	1.144 (.147)	.964 (.116)
	Low level of education	.842 (.164)	.773 (.148)	.674* (.128)
	High level of education	.865 (.168)	.833 (.128)	1.217 (.166)
	England	1.616 ** (.286)	1.884*** (.275)	.884 (.116)
	Q1	NA	.889 (.083)	1.000 (.081)
	Q2	1.051 (.106)	NA	.920 (.062)
	Q3	.805* (.088)	1.132 (.082)	NA
	Constant	.512 (.220)	.313*** (.095)	.499* (.149)
	Number of observations	2782	2782	2782
	Pseudo R2	.0173	.0479	.0184

* = *P* < .05, ** = *P* < .01, *** = *P* < .001; robust standard errors presented within parenthesis

In England, people were less likely to want a health professional such as a GP or hospital doctor (consultant) to make decisions about their treatment (0.697**) and more likely to want to make their own decisions (1.616**). Women were less likely than men to want a health professional to make decisions about their treatment (0.787*). People who disagreed more with the statement they wanted a health professional such as a GP or consultant to make decisions about their treatment (that is to a greater extent wanted to make their own decisions) were also more likely to state that they would like to be involved in organizational decision-making (1.285**). We found no significant effect of age or education. Controlling for sex, age and education, there were no significant differences between Swedish and English respondents regarding whether people were able to help make improvements in the local health service (0.943 ns). People reporting lower educational attainment were less likely to disagree with the statement (0.674*) whereas older people were more likely to disagree (1.015***). Furthermore, people who disagreed more with the statement that people are able to help make improvements were less inclined to want to be involved in organizational decision-making (0.830**).

The most pronounced country differences related to whether people wanted to be involved in decision making concerning the organisation and provision of health and social care services in the local area. The English respondents were less likely to agree with the statement (0.642**) and more likely to disagree (1.884***). Women were also more likely to want to be involved (1.294*), and the wish to be involved increased slightly with age (1.015***). However, the desire to be involved also decreased slightly with age (1.017***), and those with lower educational attainment were less likely to want to be involved (0.518**). Furthermore, people who disagreed more with the statement that they wanted to be involved (that is wanted less involvement) also believed less in people’s ability to help make in improvements in the local health service (0.684***). A reduced likelihood of wanting to make their own treatment decisions was also found among those who did not want to be involved in local service improvement (0.805*). This means that those who did not want to make individual treatment decisions also did not want to be involved in decision making about the organisation and provision of health and social care services.

## Discussion

### Patient involvement

Overall, our results support previous findings [11, 26] suggesting that many patients – 66% of the English and 70% among the Swedish respondents – prefer the physician to make health care decisions (professional-determined involvement) and that few want to take a fully active role (patient-determined involvement [39]. However, even if patients do not want to play an active role in treatment decision-making, most want physicians to take their preferences into account and to inform them of options. Not feeling properly informed about their treatment is one of the most common sources of patient dissatisfaction [22]. Furthermore, our results show that Swedish respondents when compared to the English more often wanted a health professional such as a GP or a consultant to make decisions about treatment on their behalf and were less inclined to want to make decisions themselves, even if the differences were rather small (compare [12]).

We do not know what lies behind these country differences, which for example, could be linked to level of trust or patient centeredness. Trust in the healthcare provider has been linked to a wish for a less autonomous role [40]. Data on the general level of trust in health professionals however indicates a similar level in both countries; in 2013, 89% of the English reported they trusted doctors [41] and 88% of the Swedes that they trusted healthcare staff [42]. However, in contrast to England, the level of patient-centeredness in Sweden is systematically ranked low [43] and Swedish patients have traditionally had a weak position [44]. For instance, an international evaluation in 2012 concluded that more effort is needed to ensure that patients are adequately equipped to partner with their providers [26]. Thus, the relative lack of patient empowerment, participation and partnership experience may be one reason why the Swedish respondents were more inclined to answer that they want health professionals to make decisions about their treatment. Coulter et al. [22] suggest that interventions such as patient coaching and question prompts can help empower patients together with decision aids, measures rarely used in Sweden. However, evidence suggests that patients need becoming more active partners in England as well as in Sweden as “there has been a lack of systematic progress” when it comes to patient involvement and ‘putting patients first’ [45]. However, not everyone wants to be actively involved. For example, limited experiences of healthcare (i.e. low utilization) as well as negative experiences of healthcare or of health professionals have all been linked to a low desire for involvement. Furthermore, type of illness and seriousness are linked to demand for involvement: chronic conditions and low seriousness leading to an increased demand [39]. To understand how to support an active patient style is important as patient involvement has been linked to improved health outcomes [5], and been presented as particularly important to mitigate the negative health effects of chronic conditions, now the largest cause of disability and death worldwide [46]. In relation to long-term illness the way clinicians and patients interact too often promote passivity and dependence [12] and some professionals and organizations are still threatened by the notion of active involvement [47]. In end-of-life care, active involvement is perhaps particularly challenging as not all patients want to be fully informed about their health condition. Studies indicate that a majority of patients and relatives want to leave the final decisions about medical treatment to the health professionals, although being involved in the decision-making process at the “right time” [48].

### Public involvement

Our findings also demonstrate that Swedes were more positive towards being involved in local decision making on the organisation and provision of health and social care services; 55% of Swedish respondents wanting to be involved compared to 33% among the English. This may be linked to, for example, the level of satisfaction with the current organisation of services or assumptions about the impact or effect of being involved. For instance, dissatisfaction with the current health system may lead to a greater interest in being involved in local decision-making in order to improve services. International comparisons suggest a marked difference between Sweden and England in the overall views of the health care system. In 2013, 44% of the Swedes compared to 63% of the English agreed that the health care system works well and only needed minor changes [49]. Furthermore, the design and implementation of PPI activities that fail to achieve the intended goals and do not genuinely involve people can make people less willing to be involved [9, 50]. Yet, the country difference in willingness to be involved – the Swedes expressing a greater willingness – did not correspond to a statistically significant country difference in people’s perceptions of whether they are able to help make improvements to local services, that is whether they perceive involvement has an impact and is worthwhile. Sound empirical evidence of the outcomes of public involvement is underdeveloped [51] and in our sample, only 36% of Swedish and 39% of English respondents, agreed that people are able to help make improvements to local services. This suggests that a willingness to be involved is complex, and that explanations may be sought in individual factors as well as in relation to cultural, organizational and political contexts (c.f. [47]). Willingness as well as the capacity to be involved are affected by multiple factors; beliefs about role, education, health literacy, organizational practice and culture, social norms, and policy and regulation [47]. For instance, Sweden is a country with a large public sector while having a vital civil society and high levels of social capital, measured for instance in membership of voluntary associations [52]. Participation has also been identified as a fundamental characteristic of Nordic health systems [36], largely carried out locally. Such contextual factors may have an impact on the willingness to be involved collectively. We suggest that the role of local democratic decision-making is important in explaining differences in involvement willingness we observe but this needs further investigation. In Sweden regional governments have far greater responsibility and autonomy for decisions about the organisation and provision of healthcare. In England, Central government allows very limited variation in local determination of provision and this has been curtailed over the last 5 years while cost-containment concerns and industrial relations with physicians have been the primary focus.

Nevertheless, at the individual level in our study, those who disagreed more with the statement that they wanted to be involved in local organizational decisions also believed less in people’s ability to help make improvements. While we know little about the experience or consequences (impact) of public involvement in either country [51] it is unsurprising that those who felt there was little scope for influence at a local level were less likely to want to be involved. Valid measures of the impact of involvement might illustrate the relevance of involvement and encourage participation.

### Sociodemographic characteristics

Some sociodemographic characteristics were significant. Women were less likely to want health professionals to make decisions [13] and more likely to want to be involved in organizational decisions. Regarding individual treatment decisions, we found no significant effect for age or educational attainment despite evidence of these in previous studies [11, 13, 16]. However, age both increased and decreased the likelihood of expressing a wish to be involved in organizational decisions, which may be linked to higher levels of contact with health care and time availability leading to greater desire to shape provision but amongst the more ill an inability to be involved [12, 53]. Low levels of educational attainment reduced the willingness to be involved and the likelihood to disagree that people were able to help make improvements, which may be associated with a limited experience of being involved (c.f. [54]). Thus, our results point to complex relationships between sociodemographic factors and how preferences for involvement are shaped and expressed. In fact, demographic and situational characteristics have been calculated to explain only 20% or less of the variability in preferences [11]. Yet, there was a significant relationship between a willingness to be involved in individual treatment decisions and the willingness to be involved in collective decision-making about local service provision, indicating that the willingness to be involved may be concentrated in a particular group of individuals. It is, however, important to note that there may be a difference between the individuals expressing a willingness to be involved and those who *actually participate* in involvement activities as surveys measure expressed views rather than behaviour. As many involvement activities are based on self-selection, this may reproduce social inequalities in the distribution of the benefits associated with PPI [21].

### Limitations and methodological considerations

We acknowledge some limitations to this study. As country data about PPI and preferences for involvement is not collected and accessible through e.g., the OECD Health Statistics or the Eurobarometer Surveys, we had to collect it for the purpose of this study; this was done in collaboration with Ipsos MORI in England and TNS Sifo in Sweden. Thus, this is a one-off cross-sectional measurement in two countries and we are not able to present longitudinal results. Although data were collected with structured interviews using two different techniques (telephone vs. face-to-face), it was in both cases collected via personal contact between the respondent and the collecting interviewer in contrast to using a printed or electronic survey and self-completion. In our assessment the nature of the questions was not such that the answers were affected by the slightly different collection methods. This is strengthened by the variation in which country scored highest. To ensure coherence, we excluded the results from a fourth question that was presented slightly differently in the face-to-face interviews compared to the telephone interviews. The survey questions were formulated in parallel in Swedish and in English and discussed among the first and last author (native Swedish speaker and native English speaker, respectively) with extensive experience from health service research in the two countries to ensure the questions would be as similar as possible and have the same connotations in the Swedish and English context. Although the weighting variable is not identical, it was in both countries constructed to create a sample representative of the population. Lastly, the potential explanations of the country differences we discuss need to be tested with individual-level data on e.g. trust in physicians, perceived patient-centeredness and satisfaction with the health system as well as general attitude to be involved in matters of collective concern. Research using approaches such as open-ended interviews or focus groups would support a more nuanced understanding of the interaction between healthcare utilization and experience and expectations of being involved. Such approaches might also reveal that involvement is of more interest in some aspects of healthcare rather than others.

Our discussion thus has implications for the direction of further research and for health policy and PPI practice. It is also important to note that we did not measure actual involvement, typically triggered by a concern about a particular issue such as poor quality care [55], which may also be shaped by factors we did not include in the analysis; for example experience of involvement, cognitive skills, health status or health care utilization [47]. Thus we do not know how the samples reflect consumers of healthcare services. At an aggregate level, however, we know that healthcare utilization increases with age. For instance, in Sweden those aged 65 and over constitute just below 20% of the population but account for about 55% of bed-days [56]: making this group particularly important to involve. Lastly, there may also be area effects we have not captured as opportunities and experience of involvement are shaped by the local context in both England and Sweden.

## Conclusions

There are complex patterns behind preferences for involvement and there is still little theorising on the motivation of service users to get involved [55]. Views on involvement may vary between cultures and settings [12]. In the decentralized Swedish health system, people expressed greater willingness, compared to respondents in England, to be involved in decision making regarding the organisation and provision of health and social care services in the local area; i.e. to be involved as public policy agents in decisions of a broader public interest. To what extent these preferential differences translate into differences in actual involvement and in the impact of involvement needs further exploration. In both countries, involvement policies that alter the balance between the system, the health professionals and patients (c.f. [47]) are needed as many preferred a passive role in individual decisions. Regardless of the expressed willingness to participate and the motivations that shape involvement preferences and behaviour, "patient participation in decision-making is justified on humane grounds alone" [57]. As essential is the right of both patients and citizens to be involved in service design and development that shape local health and social care priorities. More explicit evidence of the impact of involvement and greater attention to equity in the opportunities and take up of PPI are necessary to achieve both individual level benefits such as improved health and collective level benefits such as local community responsiveness and improved public health. Those not taking part in involvement activities are often those who have the most to gain from involvement in healthcare decision-making, that is those with greater healthcare needs [47]. It is thus important to recruit and provide a range of opportunities to participate to involve so called non-participants [55]. National PPI strategies that take account of the specific structural features of a health system, such as level of (de)centralization are also fundamental to increase the effectiveness of involvement rather than simply appropriating approaches used in other countries. To design health systems to promote health and well-being is integral to public health and effective PPI is an essential element of such systems [58].
